# *SLC7A11*-mediated disulfidptosis underlies the pathogenesis of proliferative diabetic retinopathy

**DOI:** 10.1186/s40662-026-00511-2

**Published:** 2026-09-26

**Authors:** Bowei Yuan, Shuilian Chen, Jiayan Liu, Tingting Yang, Feng Zeng, Rui Zhang, Zhihui Xiao, Xi Chen, Jin Qiu, Cong Wei, Zhiyan Zhou, Qiutong Pan, Yamei Lu, Jing Zhuang

**Affiliations:** 1 Affiliated Qingyuan Hospital, Guangzhou Medical University, Qingyuan People’s Hospital, B24 Yinquan Road, Qingcheng District, Qingyuan, 511500 Guangdong China; 2 State Key Laboratory of Ophthalmology, Zhongshan Ophthalmic Center, Sun Yat-Sen University, No. 7 Jinsui Road, Tianhe District, Guangzhou, 510060 China; 3 Guangdong Provincial Key Laboratory of Ophthalmology and Visual Science, No. 7 Jinsui Road, Tianhe District, Guangzhou, 510060 Guangdong China; 4 Guangdong Provincial Clinical Research Center for Ocular Diseases, No. 7 Jinsui Road, Tianhe District, Guangzhou, 510060 Guangdong China

**Keywords:** Disulfidptosis, SLC7A11, Proliferative diabetic retinopathy, Angiogenesis, Glucose deprivation

## Abstract

**Background:**

We investigated the role of disulfidptosis, a recently identified form of cell death, in the pathogenesis of proliferative diabetic retinopathy (PDR) and explore the function of the cystine/glutamate antiporter, SLC7A11, as a key mediator and potential therapeutic target.

**Methods:**

Bioinformatic analysis was used to screen disulfidptosis-related genes from public RNA sequencing datasets, which were subsequently validated in PDR fibrovascular membranes (FVMs) and vitreous humor via immunofluorescence and western blotting analysis. Human retinal microvascular endothelial cells (HRMECs) were cultured in normal or high-glucose media, and *SLC7A11* expression modulated using small interfering RNA or lentiviral overexpression. Angiogenesis was assessed via proliferation, migration, and tube formation assays. Disulfidptosis was induced via acute glucose deprivation in high-glucose-adapted HRMECs and biochemically evaluated. A streptozotocin-induced diabetic mouse model was utilized to assess *SLC7A11* expression in vivo, and therapeutic efficacy was tested through weekly intravitreal lavage with glucose-free versus glucose-replete solutions.

**Results:**

Disulfidptosis-related genes, particularly *SLC7A11*, were significantly upregulated in PDR FVMs, predominantly localizing to the vascular endothelium. In vitro, high-glucose conditions induced *SLC7A11* and vascular endothelial growth factor A expression in HRMECs, thereby promoting proliferation, migration, and tube formation. *SLC7A11* knockdown abrogated these pro-angiogenic effects, whereas overexpression exacerbated them. Critically, high-glucose-adapted cells developed a profound dependency on glucose; subsequent acute glucose deprivation triggered rapid cell death, exhibiting classic disulfidptosis features (F-actin collapse), a process that could not be rescued by ferroptosis inhibitors. In the diabetic mouse model, *SLC7A11* expression was similarly upregulated in the pathological retinal vasculature. Therapeutic vitreous lavage with a glucose-free solution significantly attenuated the progression of these pathological neovessels.

**Conclusions:**

Our results revealed a novel *SLC7A11*-mediated metabolic vulnerability in PDR. We demonstrated that exploiting this glucose dependency via localized deprivation induces disulfidptosis-like features in neovascular cells, representing a new therapeutic strategy for PDR.

**Supplementary Information:**

The online version contains supplementary material available at https://doi.org/10.1186/s40662-026-00511-2.

## Background

Diabetic retinopathy (DR) is the most frequent microvascular consequence of diabetes mellitus and leading cause of vision impairment in working-age adults across the globe [1]. Based on current epidemiological data, the number of individuals affected globally by DR is projected to increase from 103 million in 2020 to 161 million by 2045, a sharp increase that highlights a significant and worsening global-health problem [2–4]. The focus of current therapeutic mainstays, including pan-retinal photocoagulation and intravitreal anti-vascular endothelial growth factor (VEGF) agents, is on managing the effects of ischemia rather than its underlying causes. Although effective in many cases, these treatments are invasive and require repeated administration, and a significant proportion of patients exhibit incomplete response or nonresponse [5]. Importantly, these therapies do not address the foundational metabolic dysregulation and resulting metabolic inflexibility responsible for the onset and perpetuation of the disease process [6]. This drawback has created a pressing need for novel mechanistic interventions based on the core pathophysiology of DR.

DR pathogenesis is intrinsically linked to hyperglycemia-induced metabolic turmoil, which culminates in chronic oxidative stress and a low-grade inflammatory state in the retinal microenvironment [7, 8]. This toxic milieu triggers a cascade of cellular stress responses, ultimately leading to various forms of regulated cell death (RCD) in retinal cells [9]. Recent evidence implicates multiple RCD pathways in DR, including pyroptosis, necroptosis, and ferroptosis, each of which contributes to vascular leakage, inflammation, and endothelial dysfunction [10–12]. Identification of the molecular linchpins that orchestrate these cell-fate decisions, particularly within the vascular endothelium, is paramount for developing next-generation therapeutics.

Disulfidptosis, a newly identified mode of RCD, is initiated by the overabundance of intracellular cystine and other disulfide compounds [13]. This accumulation precipitates disulfide stress, promotes anomalous cross-linking of actin cytoskeleton proteins, and ultimately causes structural collapse of the cell [9]. Disulfidptosis critically depends on the cystine/glutamate antiporter, SLC7A11, which facilitates excessive cystine influx, leading to disulfide stress and actin cytoskeleton collapse. The function of *SLC7A11* in cellular homeostasis is twofold and well established [13–15]. By mediating the uptake of cystine—the rate-limiting molecule for glutathione (GSH) production—SLC7A11 underpins the cellular antioxidant defense system, providing crucial protection against oxidative stress phenomena [10, 16, 17]. This protective function is often hijacked by cancer cells to support survival and proliferation under high oxidative stress and is fundamentally linked to the prevention of ferroptosis, a form of iron-dependent RCD driven by the catastrophic accumulation of lipid peroxides [18, 19].

In the canonical anti-ferroptotic pathway, synthesis of the principal antioxidant, GSH, is dependent on cysteine, which is derived from the reduction of extracellular cystine following its transport into cells via the SLC7A11 antiporter [8]. As a critical cofactor, GSH enables GSH peroxidase 4 to neutralize toxic lipid peroxides, thereby safeguarding cellular membranes from oxidative destruction [20]. Consequently, the upregulation of *SLC7A11* expression is a well-established survival mechanism, often hijacked by cancer cells, to confer robust resistance against ferroptotic insults [20]. Paradoxically, this dependency creates a critical vulnerability. Specifically, under glucose deprivation, high levels of *SLC7A11* expression are lethal, leading to a massive influx of cystine that cannot be reduced via the now-crippled nicotinamide adenine dinucleotide phosphate (NADPH)-dependent thioredoxin system, thereby precipitating fatal disulfide stress and disulfidptosis [9, 13]. Given that the DR microenvironment is a hotbed of oxidative and endoplasmic reticulum stress [16], determining whether and how *SLC7A11* contributes to its pathogenesis is important.

In this study, we used integrated bioinformatics, patient sample analysis, advanced in-vitro cell models, and a preclinical in-vivo model to elucidate the role of *SLC7A11* in proliferative DR (PDR). We demonstrated that *SLC7A11* is specifically overexpressed in the retinal endothelium of patients with PDR and is essential for the pro-angiogenic activities of endothelial cells under hyperglycemic conditions. Critically, we revealed that this *SLC7A11* dependency can be therapeutically exploited by inducing localized glucose restriction, which selectively triggers disulfidptosis in the aberrant vasculature, leading to disease attenuation. Our findings uncover a fundamental metabolic vulnerability in PDR and present a novel therapeutic paradigm for its treatment.

## Methods

### Ethics statement

This study was conducted under full ethical adherence. All procedures involving animals complied with the Association for Research in Vision and Ophthalmology Statement for the Use of Animals in Ophthalmic and Vision Research and were approved by the Animal Ethics and Experimentation Committee of the Affiliated Qingyuan Hospital (No. LAEC-202410-005). The protocol for using human patient samples was approved by the Ethical Committee of the Qingyuan Affiliated Hospital of Guangzhou Medical University (No. IRB-2024-194). All patients provided written informed consent before enrollment, and the specimens were managed according to the principles of the Declaration of Helsinki.

### Data analysis for single-cell and mRNA sequencing

Bioinformatic analysis was conducted on two datasets sourced from the Gene Expression Omnibus (GEO) database. For differential expression analysis, bulk RNA sequencing (RNA-seq) data (GSE102485) obtained from the fibrovascular membranes (FVMs) of patients with PDR (n = 20) and healthy controls (n = 3) were processed using the limma R package, with a significance cutoff of adjusted *P* value < 0.05 and |log_2_(fold change [FC])| > 1 [21]. For single-cell transcriptomic profiling, single-cell RNA-seq (scRNA-seq) data obtained from patients with PDR (GSE165784) were analyzed with the Seurat R package (from 5 patients with PDR, as reported in the original GEO dataset). The analytical workflow included quality control (retaining cells with 200–5,000 genes; < 20% mitochondrial DNA), data normalization and scaling, and dimensionality reduction via principal component analysis. Cell populations were clustered using the Louvain algorithm and subsequently annotated by cell type using the SingleR package and a set of canonical markers.

### Patient samples

Vitreous humor and epiretinal FVMs were acquired during vitrectomy. Samples from patients diagnosed with PDR constituted the experimental group. Control samples were procured from patients undergoing surgery for nondiabetic pathologies, such as epiretinal membranes. All specimens were placed on ice immediately after excision and subsequently stored at − 80 °C (12 patients with PDR and 12 nondiabetic controls).

### Cell culture

Human retinal microvascular endothelial cells (HRMECs) (CSC, Kirkland, WA, USA) were cultured in glucose-free endothelial cell medium (ECM) (1001-GF; ScienCell, Carlsbad, CA, USA) with 10% fetal bovine serum (FBS; BIOEXPLORER, Boulder, CO, USA), and the media refreshed every two days. Upon reaching 85%–90% confluence, the HRMECs were subcultured at a 1:3 ratio. To simulate diabetic conditions in vitro, the HRMECs were treated with 30 mM anhydrous glucose (Meilunbio, LiaoNing, China) in ECM for 72 h; controls received 5 mM glucose and 25 mM mannitol (to control the potential effects of hyperosmolarity independent of glucose metabolism). Cell generations 8–10 were used for follow-up analysis.

### Induction of disulfidptosis and pharmacological validation

Disulfidptosis induction was initiated by replacing the high-glucose (HG) culture medium. Following aspiration of the existing medium and a single wash with phosphate-buffered saline (PBS), the cells were incubated for 8 h or 16 h in glucose-free ECM fortified with 10% dialyzed FBS and 1% penicillin–streptomycin.

For the ferroptosis inhibition experiment, cells were pretreated with the specific ferroptosis inhibitor, ferrostatin-1 (Ferr-1), for 24 h before and during the glucose deprivation period. For the ferroptosis-positive control, cells cultured in a standard HG medium were treated with the ferroptosis inducer, erastin, with or without Ferr-1 co-treatment for 24 h [22].

### Lentiviral overexpression and small interfering RNA knockdown

For overexpression, the human *SLC7A11* coding sequence was cloned into the pLV-Puro lentiviral vector. To establish stable cell lines, HRMECs were first transduced with lentivirus produced in HEK293T cells, after which a selection process was carried out using 2 μg/mL puromycin. For knockdown, HRMECs were transfected with *SLC7A11*-specific small interfering RNAs (siRNAs; 50 nM) or a nontargeting control siRNA (GenePharma, Shanghai, China) using Lipofectamine RNAiMAX (Invitrogen, Waltham, MA, USA). Efficacy was assessed at 48–72 h after transfection. The sequences of *SLC7A11* siRNA (human) were 5′-GAAUCUUCAUCUCUCCUAA-3′ (forward) and 5′-UUAGAAGAGAUGAAGAUUC-3′ (reverse).

### mRNA sequencing and bioinformatic analysis

The transcriptomes of *SLC7A11* overexpression (*SLC7A11*-OE) and control cells were analyzed via mRNA sequencing. Total RNA was initially extracted using TRIzol reagent, and its quality subsequently assessed on a 2100 Bioanalyzer (Agilent Technologies, Santa Clara, CA, USA). After the ribosomal RNA was depleted, 50 ng total RNA was used to prepare directional sequencing libraries with the ScriptSeq v2 RNA-seq Library Preparation Kit (Epicentre Biotechnologies, Madison, WI, USA). Libraries with an average size distribution of 300–500 bp were sequenced using an Illumina NextSeq 500 system (Illumina, San Diego, CA, USA). The raw sequencing data obtained from this analysis have been submitted to the NCBI BioProject database and are accessible under the accession number PRJNA1329159.

### Western blotting assay

For protein analysis, total protein was extracted from cells and human vitreous tissue using radioimmunoprecipitation assay buffer supplemented with protease inhibitors. To prepare vitreous humor samples, they were initially clarified via centrifugation at 12,000×*g* for 10 min at 4 °C, and the resulting supernatant was then collected. The total protein concentration of all samples was quantified using a bicinchoninic acid assay to ensure equal loading for subsequent analysis (12 PDR and 12 controls, quantified in triplicate).

For each sample, 10 μg total protein was resolved on sodium dodecyl sulfate–polyacrylamide gel electrophoresis gels and then transferred to polyvinylidene fluoride membranes. The membranes were blocked with 5% nonfat milk for 3 h at room temperature (23–25 °C). Following blocking, the membranes were incubated with primary antibodies (see Additional File 1 Supplementary Table S2) overnight at 4 °C. The next day, the membranes were washed and incubated with the appropriate horseradish peroxidase-conjugated secondary antibody (anti-rabbit [#S0001] or anti-mouse [#S0002]; Affinity Biosciences, Cincinnati, OH, USA) for 2 h at room temperature (23–25 °C). To confirm equal protein loading, the membranes were stripped and re-probed for the loading control, β-tubulin. Finally, the relative intensity of the protein bands was quantified using ImageJ software (National Institutes of Health, Bethesda, MD, USA) [23]. The total protein concentration in the vitreous samples was 0.683 µg/µL (ranging from 0.212–0.828 µg/µL).

### Immunofluorescence staining assay

Immunofluorescence staining experiments were performed on epiretinal FVMs derived from patients with PDR and nondiabetic controls, as well as on mouse retinal tissues (flat mounts and cryosections). The samples were processed for fluorescent imaging following a 1-h fixation in 4% paraformaldehyde (PFA) and 30 min of blocking with 5% bovine serum albumin (BSA) (Beyotime, Beijing, China). For immunostaining, the samples were sequentially incubated with primary antibodies (Additional File 1 Supplementary Table S2, overnight at 4 °C) and an Alexa Fluor 555 anti-rabbit IgG secondary antibody (1:500, Cat No.: 4413; Cell Signaling Technology, Danvers, MA, USA). Nuclei were counterstained with 4′,6-diamidino-2-phenylindole (1 µg/mL) for 5 min at room temperature (23–25 °C) in the dark. In separate preparations for structural analysis, the fixed cells were co-incubated with Deep Red Actin Tracking Stain (A57245; Thermo Fisher Scientific, Waltham, MA, USA) and CellMask Green Plasma Membrane Stain (C37468; Thermo Fisher Scientific) for 45 min at 37 °C in the dark. A Zeiss 900 fluorescence microscope (Zeiss, Oberkochen, Germany) was used to capture images.

### Cell viability assay

The effect of *SLC7A11* overexpression and ferroptosis on cell proliferation was quantified using the Cell Counting Kit-8 (CCK-8) assay (BS350A; Biosharp, ON, Canada). Assays were conducted after 72 h of HG culture for the HG group, or at 24 h after transfection for the *SLC7A11* overexpression or knockdown experiments. Following a 3-h incubation with the CCK-8 reagent at 37 °C, cell viability was determined by measuring the optical density at 450 nm (performed in three independent biological replicates, with three technical replicates per experiment).

### Wound healing and tube formation assays

For the wound healing assay, HRMECs were cultured in six-well plates until they reached 90% confluence. The growth medium was then replaced with serum-free ECM. Once the cells formed a complete monolayer (100% confluence), a sterile 200 μL pipette tip was used to create a linear scratch. Wound closure was monitored and imaged at specific time points using a light microscope. The scratch area was subsequently quantified using ImageJ.

For the tube formation assay, 96-well plates were initially coated with 50 μL/well Matrigel and incubated at 37 °C for 30 min to allow solidification. Following this, HRMECs were seeded onto the Matrigel layer in fresh medium. After a 12-h incubation period, the formation of capillary-like networks was observed and photographed under a light microscope. Angiogenic activity was quantified by measuring the average number of nodes, junctions, and meshes from multiple fields per sample using ImageJ [24] (performed in three independent biological replicates, with three technical replicates per experiment).

### Functional enrichment analysis

To elucidate the functional consequences of *SLC7A11* overexpression, Gene Ontology pathway enrichment analysis was conducted. The analysis was performed on the set of differentially expressed genes (DEGs) with a *P* value < 0.05 and |log_2_FC| > 1 when comparing *SLC7A11*-OE and control cells. Using the clusterProfiler package in R, we performed an overrepresentation test against the Gene Ontology domains (biological processes, cellular components, and molecular functions). A false discovery rate correction was applied, and terms with a Benjamini–Hochberg-adjusted *P* value < 0.05 were deemed significantly enriched.

### Experimental animal design

A cohort of C57BL/6J mice, aged between six and eight weeks and with an equal representation of both sexes, was randomly segregated into nondiabetic control and diabetes mellitus experimental groups (12 mice per group [nondiabetic control and diabetic, balanced sex]). Briefly, this protocol consisted of administering streptozotocin (55 mg/kg; Sigma-Aldrich, St. Louis, MO, USA), prepared in sterile 0.05 M citrate buffer (pH 4.5), via intraperitoneal injection daily for five successive days. Control animals were administered with the citrate buffer vehicle exclusively. To monitor glycemic status, non-fasting whole blood was sampled from the tail veins of mice at four distinct time points: 7 days, 1 month, 2 months, and 4 months after the initial injection. Mice were operationally defined as diabetic and included in the experimental group only if their blood glucose levels consistently exceeded the threshold of 250 mg/dL across all measurement intervals. Vitreous lavage was performed as described in the “Vitreous lavage and exchange in DR mice” section. At the experimental endpoint, the mice were humanely sacrificed via CO_2_ asphyxiation according to the American Veterinary Medical Association guidelines for euthanasia. Mouse eyes were immediately enucleated for retinal analysis (including flat mounts, cryosections, and optical coherence tomography [OCT]).

### OCT and OCT angiography (OCTA) imaging analysis

Simultaneous fundus imaging was performed using a spectral-domain OCT system (VG200C; Intalight, Henan, China) equipped with an 18-mm telecentric lens. The image acquisition process incorporated two separate profiles: a widefield scanning protocol and repeated three B-scan mode. Resolution in the en face plane was maintained at an isotropic level. Data generated from the repeated scanning mode were subsequently processed to yield OCT and OCTA information. A customized algorithm employing standard deviation and mean averaging was applied to these datasets to achieve en face segmentation. The retinal average and proliferative membrane thickness difference values were quantified as the difference in hyperreflective proliferative membrane thickness between pre- and post-treatment using ImageJ.

### Fluorescein fundus angiography

Following the induction of anesthesia in C57BL/6J mice via isoflurane inhalation, fluorescein fundus angiography was performed. The procedure involved a single intraperitoneal bolus injection of 0.1 mL sodium fluorescein (Fluorescite; Alcon, Fort Worth, TX, USA). Dynamic vascular perfusion within the retina was visualized using a scanning laser ophthalmoscope (Saris Multi-modal Ophthalmic Imaging System for Animals; Robotrak Technologies Company, Jiangsu, China) configured with a 75° field of view. Image acquisition commenced concurrently with the administration of fluorescein dye, with videos recorded at a rate of 10 frames per second.

### Retinal cryosection staining and FVM immunofluorescence assay

For immunofluorescence staining, the cryosections, FVMs, and cells cultured on glass coverslips were fixed. The cryosections were already fixed, whereas the cultured cells were fixed for 10 min in 4% PFA at room temperature (23–25 °C). All samples were permeabilized and blocked for 30 min using a solution composed of PBS, 1% BSA, and 0.2% Triton X-100. Thereafter, the samples were incubated with the relevant primary antibodies (Additional File 1 Supplementary Table S2). The fractal dimension of the F-actin cytoskeleton was quantified from binarized immunofluorescence images using the box-counting method implemented in the FracLac plugin for ImageJ.

### Vitreous lavage and exchange in DR mice

To directly manipulate the metabolic microenvironment of the vitreous cavity in vivo, weekly vitreous lavage and exchange procedures were performed for four consecutive weeks. Following the induction of anesthesia in C57BL/6J mice via isoflurane inhalation, mouse pupils were dilated using 1% tropicamide eye drops. Two self-sealing sclerotomies were created approximately 1.0 mm posterior to the limbus using a sterile 34G needle, typically in the superotemporal and inferotemporal quadrants.

A 34G infusion cannula, connected via tubing to a reservoir of the irrigating solution, was inserted through one of the sclerotomies. The contralateral sclerotomy was left open to serve as a passive outflow port, allowing replacement of the native vitreous humor. Intraocular pressure was maintained at a stable level by setting the height of the infusion bottle to 30 cmH_2_O above the eye (Additional File 1 Supplementary Fig. 1a). The operation part of this experiment refers to previous literature [25, 26].

DR mice were randomly assigned to two groups: the glucose deprivation and control groups. In the glucose deprivation group, the vitreous cavity was perfused with glucose-free balanced salt solution (BSS; Alcon). In the control group, the vitreous cavity was perfused with BSS supplemented with 30 mmol/L glucose (8 mice per group: glucose-free vs. glucose-replete). The lavage was maintained for 90 s to ensure the adequate exchange of vitreal contents. This procedure was repeated once every seven days for four weeks.

### Intracellular GSH, free sulfhydryl, and NADP/NADPH assays

After treating the triplicate samples of 1 × 10^6^ cells under different conditions, we performed intracellular GSH, free sulfhydryl, and NADP/NADPH assays (Beyotime, China), according to the manufacturer’s instructions.

### Statistical analysis

All data were presented as mean ± SD or SEM, depending on the specific assay, to reflect biological variability or the precision of the estimate, respectively, from at least three independent experiments. Before comparing means, the data were assessed for adherence to statistical assumptions. Specifically, the homogeneity of variance was evaluated using Levene’s test, and normality of the distribution was assessed with the Shapiro–Wilk test. For normally distributed data meeting the assumption of equal variance, comparisons between two groups were performed using a two-tailed Student’s *t*-test. Analysis of variance was used to compare more than two groups. When the assumptions for parametric testing were not met, appropriate nonparametric equivalents were used. Across all analyses, a two-sided *P* value < 0.05 was established as the criterion for statistical significance.

## Results

### Bioinformatic profiling identifies *SLC7A11* as a key endothelial-specific disulfidptosis-related gene in PDR

**Fig. 1:**
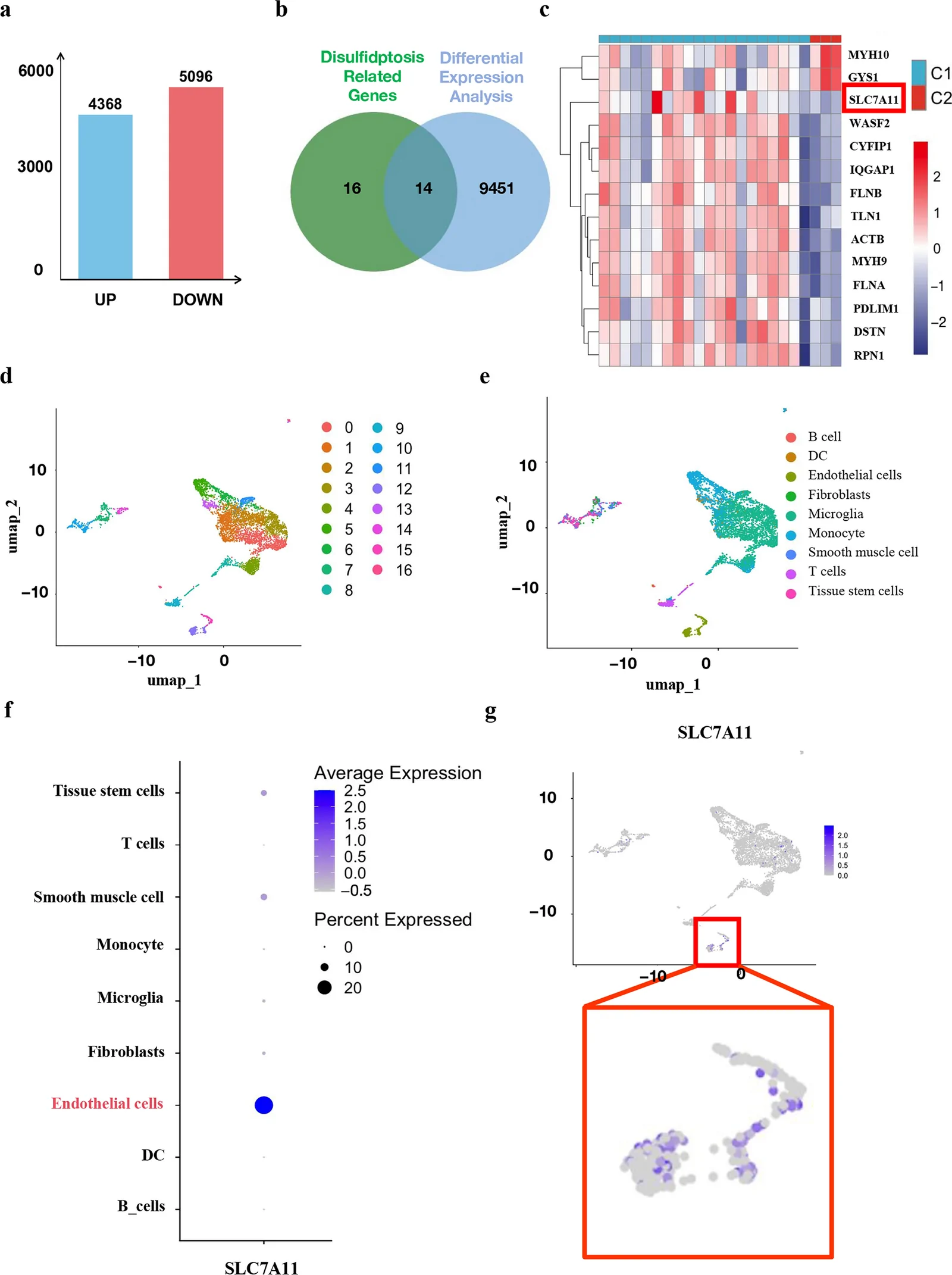
*SLC7A11* is prominently expressed in endothelial cells of proliferative diabetic retinopathy (PDR) fibrovascular membranes.** a** Bar plot showing differentially expressed genes (DEGs) in fibrovascular membranes from patients with PDR versus healthy controls, based on bulk RNA-sequencing analysis of GSE102485 dataset. **b** Venn diagram showing the intersection of DEGs from (**a**) and a curated list of disulfidptosis-related genes (DRGs), which identified 14 candidate genes. **c** Heatmap displaying expression of the 14 candidate DRGs, demonstrating the clear segregation of PDR and control samples. **d** Uniform Manifold Approximation and Projection (UMAP) plot of the PDR single-cell RNA sequencing dataset (GSE165784) showing 9 distinct cell clusters identified from 7,156 retinal cells. **e** Annotation of the cell clusters from (**d**), identifying cell types, including endothelial, Müller, and various immune cells. **f** Dot plots showing the expression distribution of key DRGs across the different cell clusters. **g** Plot highlighting the specific and prominent expression of *SLC7A11* in the endothelial cell cluster. DC, dendritic cell

We initially analyzed a bulk RNA-seq dataset (GSE102485) to systematically investigate the role of disulfidptosis in PDR. We identified 9,464 DEGs between PDR and healthy control FVMs (Fig. 1a). Cross-referencing these DEGs with a list of known disulfidptosis-related genes (DRGs) yielded 14 candidates with significantly altered expression in PDR (Fig. 1b, Additional File 1 Table S1). A heatmap of these 14 DRGs clearly distinguished the PDR and control samples (Fig. 1c).

We analyzed a PDR scRNA-seq dataset (GSE165784) to pinpoint the cellular origins of these expression changes. Unbiased clustering of 7,156 retinal cells identified 9 distinct cell types, including endothelial, Müller, and various immune cells (Fig. 1d, e). Strikingly, among the key DRGs identified, *SLC7A11* showed the highest and most endothelial-specific expression pattern (Fig. 1f, g), implicating its primary role in vascular pathology.

### *SLC7A11* expression is markedly upregulated in the proliferative membrane and vitreous humor of patients with PDR

**Fig. 2:**
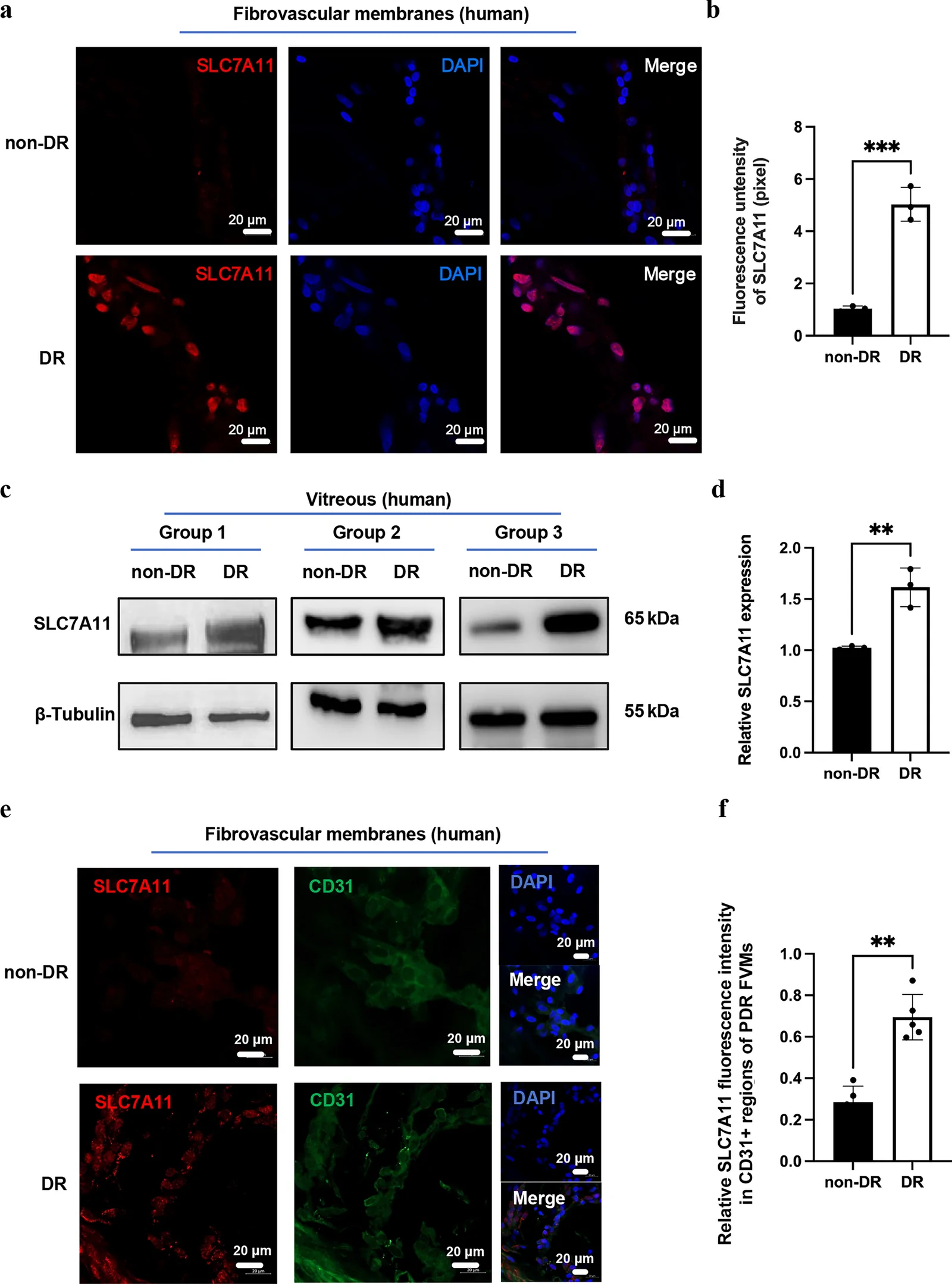
*SLC7A11* is upregulated in proliferative diabetic retinopathy (PDR) fibrovascular membranes (FVMs) and colocalizes with endothelial cell marker CD31.** a** Representative immunofluorescence images of FVMs derived from patients with PDR and epiretinal membranes obtained from nondiabetic controls. Strong SLC7A11 staining (red) was observed in PDR membranes but remained virtually undetectable in control membranes. Scale bars = 20 μm. **b** Bar graph showing quantification of the immunofluorescent signals in (**a**). **c** Western blotting analysis of soluble SLC7A11 in vitreous humor samples derived from patients with PDR (DR) and nondiabetic controls (non-DR). Three representative pairs (Groups 1–3) of the total samples are shown, demonstrating the consistent upregulation of *SLC7A11* expression in the DR group. β-Tubulin was used as a loading control. **d** Bar graph showing the quantification of western blot bands from (**c**), indicating significantly elevated levels of SLC7A11 in the vitreous of patients with PDR compared with those in controls. **e** Representative immunofluorescence co-staining of the PDR epiretinal membrane. The image shows that intense SLC7A11 staining exclusively colocalizes with the endothelial cell marker, CD31, within microvascular structures. Scale bars = 20 μm. **f** Quantification of relative SLC7A11 fluorescence intensity in CD31^+^ areas of PDR FVMs. DAPI, 4′,6-diamidino-2-phenylindole. **P < 0.01, ***P < 0.001

Based on our bioinformatic findings, we validated *SLC7A11* expression at the protein level in human clinical samples. Immunofluorescence staining of PDR-associated FVMs revealed high *SLC7A11* expression. In contrast, *SLC7A11* expression was virtually undetectable in the epiretinal membranes of the non-DR group (Fig. 2a, b). This endothelial-specific upregulation was further corroborated by the western blotting analysis results of the vitreous humor, which showed significantly higher levels of soluble SLC7A11 in samples derived from patients with PDR than in those from nondiabetic controls (Fig. 2c, d). These data confirm that SLC7A11 is a key feature of the pathological endothelium in human PDR. In epiretinal FVMs derived from patients with DR, double immunofluorescence staining revealed that strong *SLC7A11* expression was specifically colocalized within CD31-positive vascular endothelial cells (Fig. 2e, f). Such endothelium-specific *SLC7A11* overexpression highlights the critical role of metabolic reprogramming in the microvascular pathology of DR [32].

### HG drives *SLC7A11* expression and the pro-angiogenic phenotype in HRMECs

**Fig. 3:**
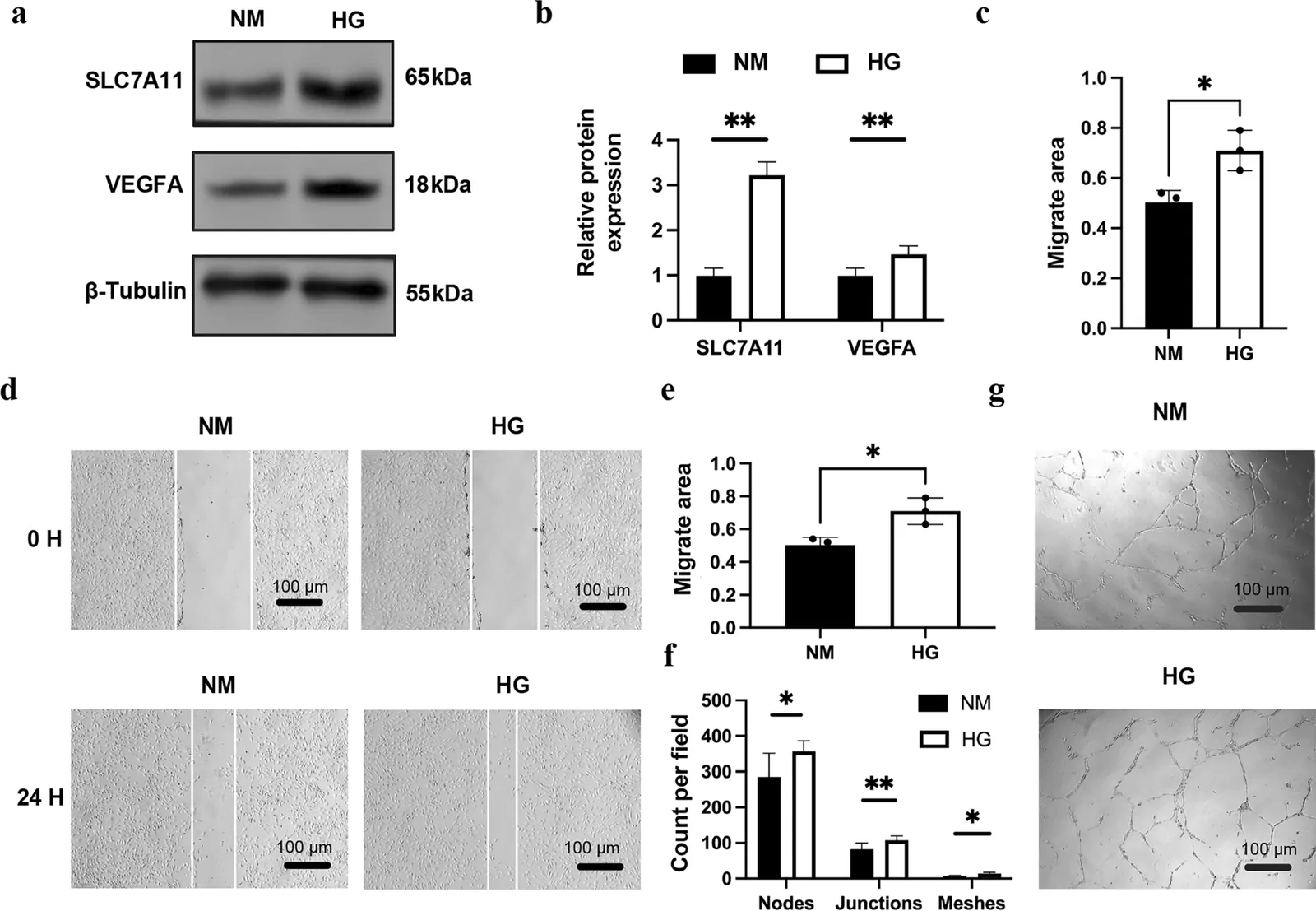
High glucose (HG) promotes proliferation, migration, and tube formation in human retinal microvascular endothelial cells (HRMECs). **a**, **b** Western blotting analysis and histogram demonstrating that HG treatment significantly upregulated the protein levels of SLC7A11 and vascular endothelial growth factor A (VEGFA) in HRMECs compared with those under normal medium (NM) conditions. **c** Cell Counting Kit-8 assay results showing that HG treatment significantly enhanced the proliferation of HRMECs. **d**, **e** Representative images and quantification of the wound healing assay, indicating that HG treatment significantly accelerated the migration of HRMECs. **f**, **g** Representative images and quantification of the tube formation assay performed on Matrigel, showing that HG-treated HRMECs formed significantly more capillary-like structures. Scale bars = 100 μm. * P < 0.05, **P < 0.01

To model the effect of hyperglycemia in vitro, we cultured HRMECs in HG medium. HG treatment significantly upregulated the expression of both *SLC7A11* and the key angiogenic factor, vascular endothelial growth factor A (*VEGFA*), at the protein level (Fig. 3a, b). Functionally, HG-treated HRMECs exhibited significantly enhanced proliferation, migration (wound healing), and capillary-like tube formation on Matrigel compared with cells grown in normal medium (Fig. 3c–g).

**Fig. 4:**
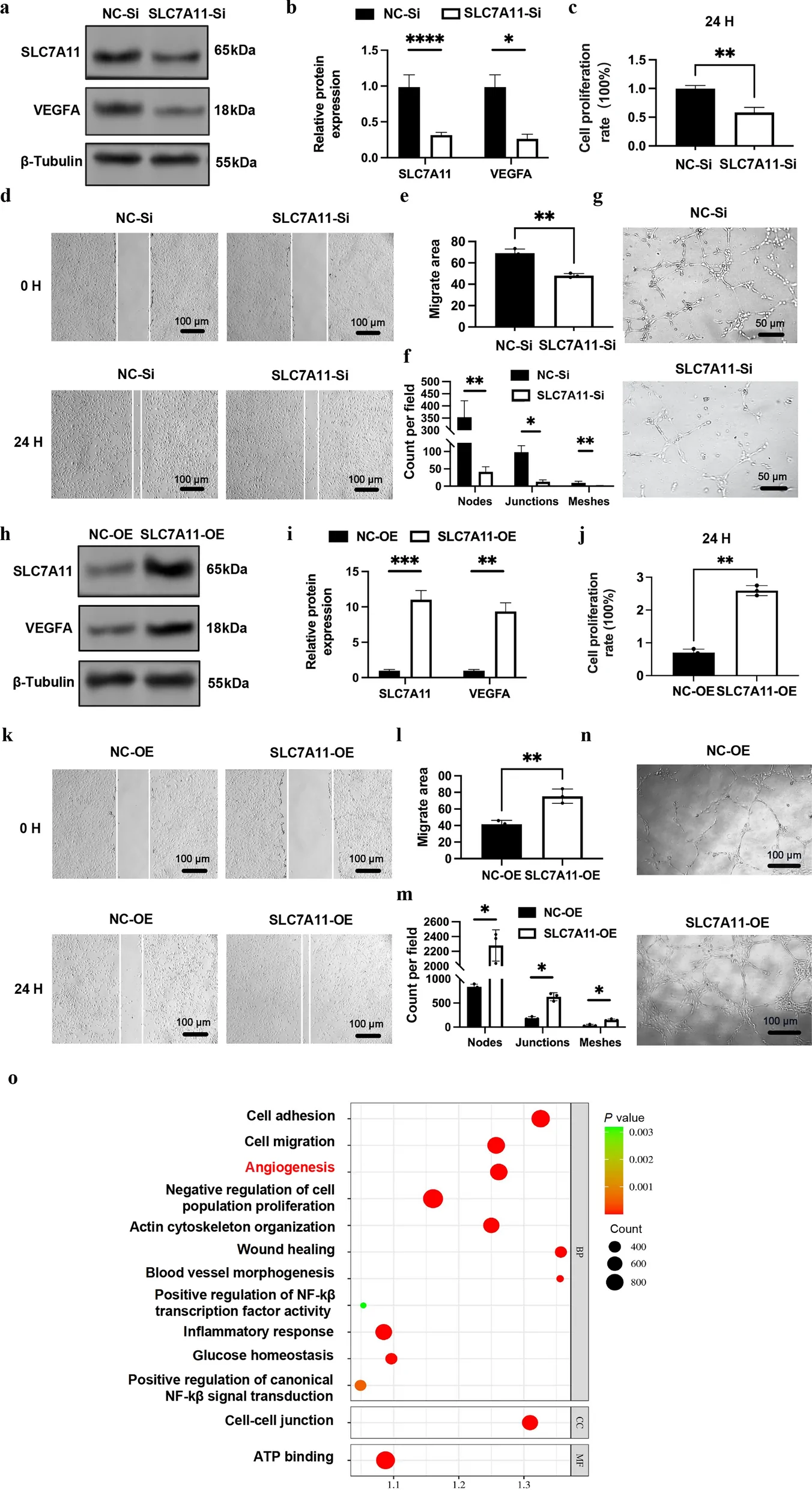
*SLC7A11* knockdown inhibits while overexpression promotes angiogenic functions. **a**, **b** Western blotting analysis and histogram confirming the efficient small interfering RNA (siRNA)-mediated knockdown of *SLC7A11* and concurrent reduction of vascular endothelial growth factor A (VEGFA) levels in human retinal microvascular endothelial cells (HRMECs) under high-glucose conditions. **c** Cell Counting Kit-8 (CCK-8) assay results showing that *SLC7A11* knockdown significantly inhibited cell proliferation compared with that in the negative control (NC) siRNA group (24 h). **d**, **e** Representative images and quantification of the wound healing assay, demonstrating a significantly decreased migration rate in *SLC7A11*-knockdown (*SLC7A11*-Si) HRMECs at 24 h. **f**, **g** Representative images and quantification of the tube formation assay, showing a significant reduction in the number of nodes, junctions, and meshes formed by *SLC7A11*-Si HRMECs. **h**, **i** Western blotting analysis and histogram confirming the successful establishment of a stable *SLC7A11*-overexpressing (*SLC7A11*-OE) HRMEC line via lentiviral transduction, which simultaneously upregulated *VEGFA* expression. **j** CCK-8 assay results demonstrating that *SLC7A11*-OE significantly promoted cell proliferation at 24 h. **k** Representative images of the wound healing assay. **l** Quantification of wound closure from (**k**), showing that *SLC7A11* overexpression markedly enhanced the migratory capacity of HRMECs. **m** Representative images of the tube formation assay. **n** Quantification of the capillary-like networks in (**m**), showing a significant increase in the number of nodes, junctions, and meshes in *SLC7A11*-OE cells. **o** Gene Ontology enrichment analysis results revealing that genes differentially expressed upon *SLC7A11* overexpression were significantly enriched in pathways related to “angiogenesis,” “cell migration,” and “wound healing.” Scale bars = 50 μm (**g**), 100 μm (**d**, **k**, **n**). BP, biological process; CC, cellular component; MF, molecular function; NC-Si, negative control siRNA; NC-OE, negative control overexpression. *P < 0.05, **P < 0.01, ***P < 0.001

To elucidate the role of *SLC7A11* in the pro-angiogenic phenotype under HG conditions, we conducted complementary loss- and gain-of-function experiments in HRMECs. For the loss-of-function experiment, siRNA-mediated knockdown was employed; three siRNAs were screened, with the most effective one robustly reducing SLC7A11 and VEGFA levels (Fig. 4a, b). This knockdown significantly inhibited cell proliferation (Fig. 4c), decreased the migration rate at 24 h (Fig. 4d, e), and reduced the number of nodes, junctions, and meshes in tube formation assays compared with those in controls (Fig. 4f, g), confirming *SLC7A11* as a critical driver of HG-induced pro-angiogenic activity. Conversely, for the gain-of-function experiment, stable *SLC7A11*-OE was achieved using a lentiviral vector, resulting in increased levels of SLC7A11 and VEGFA (Fig. 4h, i). *SLC7A11*-OE markedly enhanced proliferation at 24 h (Fig. 4j), accelerated wound closure (Fig. 4k, l), and promoted more complex capillary-like networks with an increased number of nodes, junctions, and meshes (Fig. 4m, n). Transcriptomic analysis further revealed that *SLC7A11*-OE activated gene programs enriched in angiogenesis, cell migration, wound healing, and blood vessel morphogenesis (Fig. 4o). Collectively, these assays demonstrated that *SLC7A11* potently drives a coordinated pro-angiogenic program in retinal endothelial cells under hyperglycemic conditions.

### HG adaptation sensitizes retinal endothelial cells to *SLC7A11*-dependent disulfidptosis induced by glucose deprivation

**Fig. 5:**
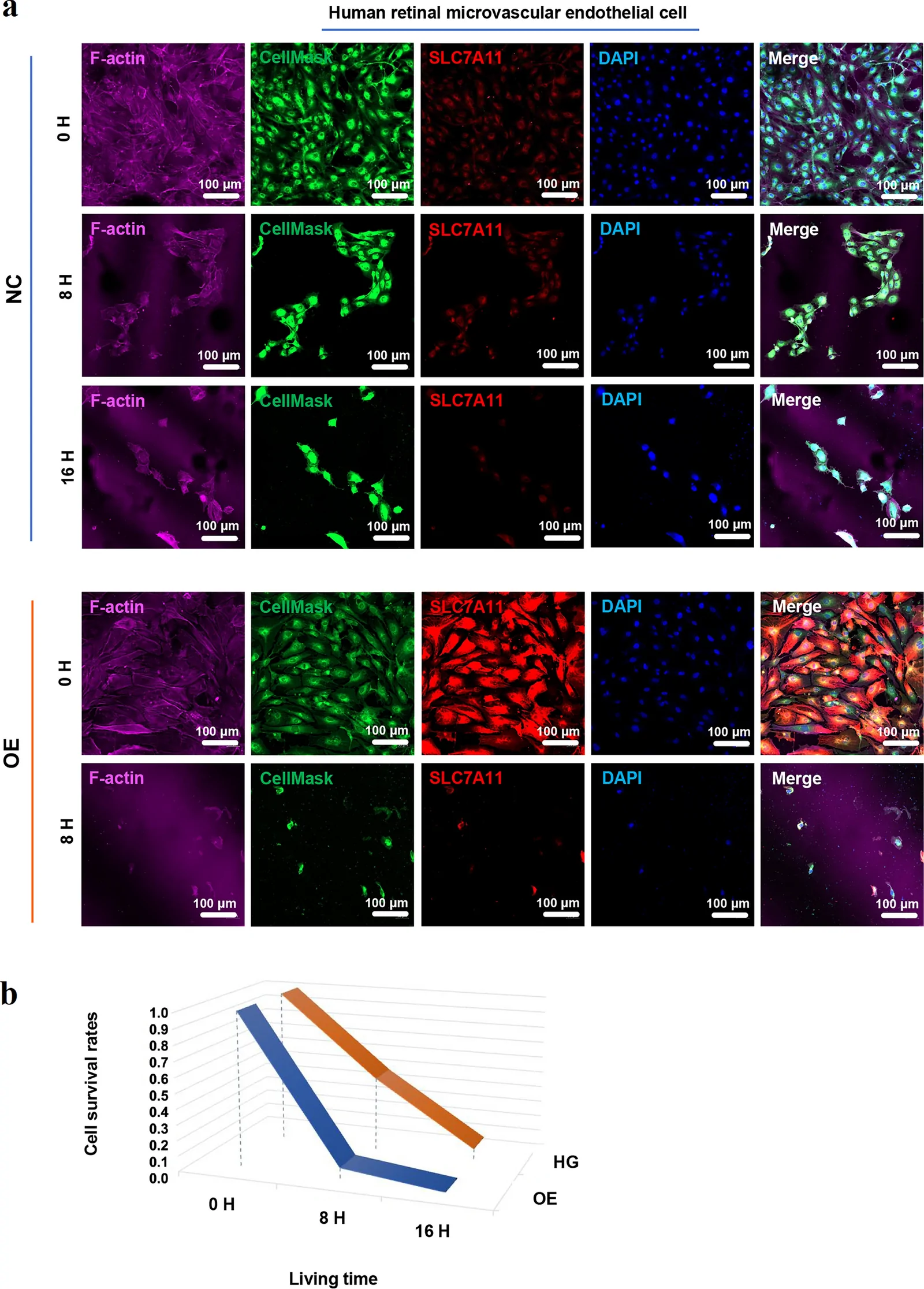
*SLC7A11* overexpression sensitizes high-glucose (HG) adapted human retinal microvascular endothelial cells to disulfidptosis upon glucose withdrawal. **a** Immunofluorescence images of control (NC) and *SLC7A11*-overexpressing (OE) cells pre-adapted to HG levels and subsequently switched to a glucose-free medium. Both groups show the hallmarks of disulfidptosis, such as cell shrinkage and F-actin cytoskeleton collapse, which were accelerated and exacerbated in *SLC7A11*-OE cells. Scale bars = 100 μm. **b** Cell survival assay quantifying the profound and rapid loss of viability in *SLC7A11*-OE cells compared with that in NC cells within 8 h of glucose withdrawal. DAPI, 4′,6-diamidino-2-phenylindole

Given that *SLC7A11* is significantly upregulated in pathological retinal endothelial cells in PDR and drove their pro-angiogenic activity under hyperglycemic conditions, as well as the fact that *SLC7A11*-mediated disulfidptosis was identified as a form of cell death inducible by glucose deprivation, we aimed to explore whether this *SLC7A11* dependency creates a unique metabolic vulnerability in HG-adapted retinal endothelial cells. We hypothesized that cells highly expressing *SLC7A11* following chronic HG adaptation would become extremely susceptible to *SLC7A11*-mediated disulfidptosis upon acute glucose deprivation, culminating in rapid cell death. To test this hypothesis, we compared negative control and *SLC7A11*-OE cells—both pre-adapted to HG conditions—after switching them to a glucose-free medium. The results were striking: immunofluorescence analysis revealed that while both groups exhibited morphological changes consistent with those described for disulfidptosis, including cell shrinkage and F-actin cytoskeleton collapse [24], the cytotoxic effect was dramatically accelerated and exacerbated in *SLC7A11*-OE cells (Fig. 5a). This qualitative observation was substantiated by a cell survival assay, which provided quantitative evidence of a profound and rapid loss of viability in *SLC7A11*-OE cells within 8 h—a rate significantly faster than that in the control cells (Fig. 5b).

**Fig. 6:**
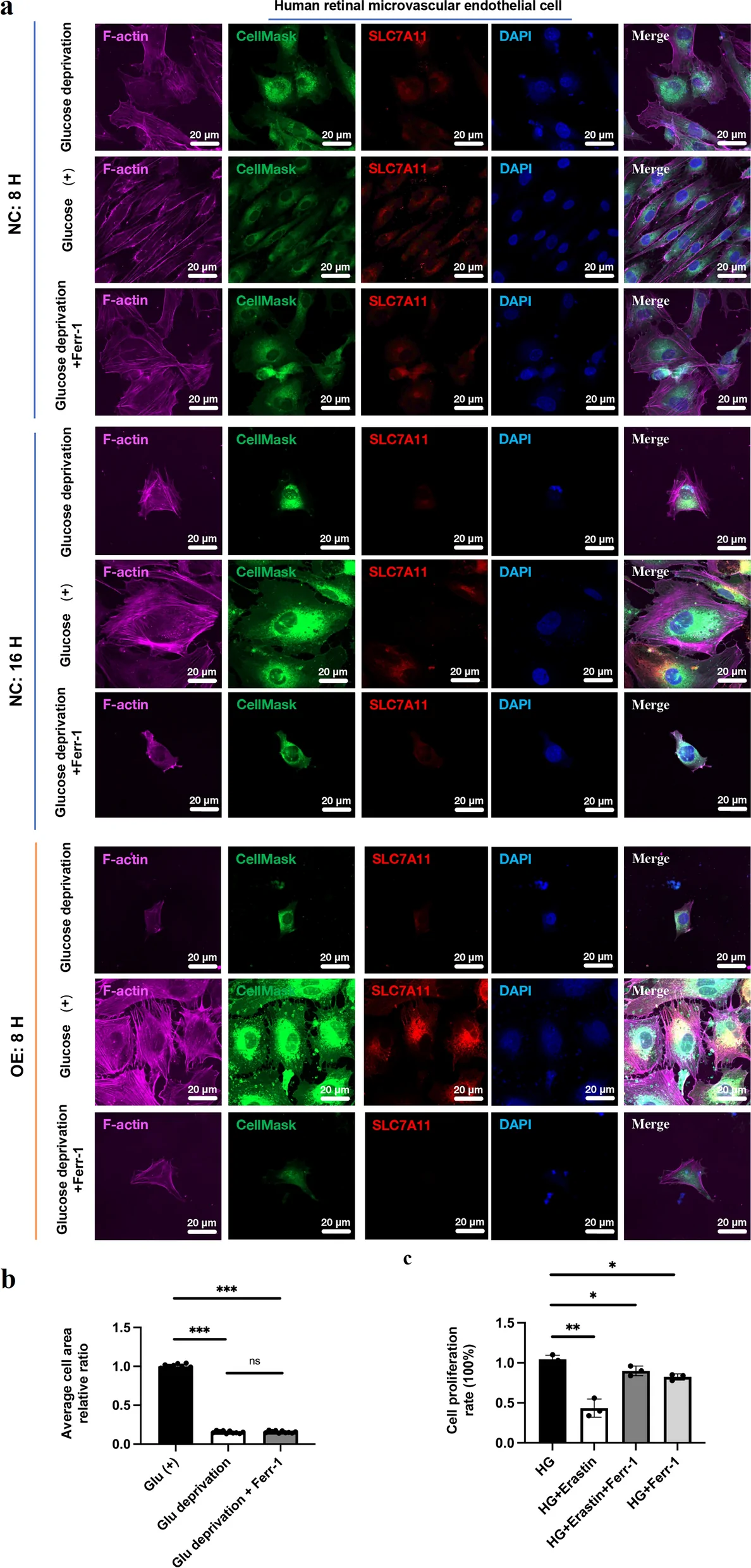
Glucose deprivation-induced death in high-glucose (HG) adapted cells is ferroptosis-independent but disulfidptosis-related. **a** Cell viability assay showing that the specific ferroptosis inhibitor, ferrostatin-1 (Ferr-1), failed to rescue the cell death induced by glucose deprivation in HG adapted cells. Scale bars = 20 μm. **b** Histogram showing the average cell area in different groups. **c** Positive control experiment showing that Ferr-1 reversed cell death induced by the canonical ferroptosis inducer, erastin. DAPI, 4′,6-diamidino-2-phenylindole; NC, normal control; OE, overexpression. *P < 0.05, **P < 0.01, ***P < 0.001; ns, not significant

Next, we sought to precisely identify the cell death modality responsible for the observed F-actin collapse. To distinguish this process from ferroptosis, we tested the effect of the specific ferroptosis inhibitor, Ferr-1. Crucially, co-treatment with Ferr-1 failed to rescue the cell death induced by glucose withdrawal. The inhibitor conferred no survival benefit, as the cells exhibited the same severe F-actin collapse and rounding morphology as those of the untreated group (Fig. 6a, b). To confirm the efficacy of the inhibitor and validate this negative result, we demonstrated that Ferr-1 completely reversed cell death induced by the canonical ferroptosis inducer, erastin (Fig. 6c).

Together with the observed *SLC7A11* upregulation and F-actin remodeling, these findings indicate that the cell death observed is driven by a ferroptosis-independent mechanism. Therefore, our results are consistent with the features of disulfidptosis. This finding revealed that chronic hyperglycemia induced a state of metabolic inflexibility, priming retinal endothelial cells for disulfidptosis upon acute nutrient withdrawal in a manner directly dependent on the cellular level of SLC7A11.

### DR mouse model reveals upregulated *SLC7A11* expression in pathological vasculature and the efficacy of glucose-free irrigation in attenuating membrane proliferation

**Fig. 7:**
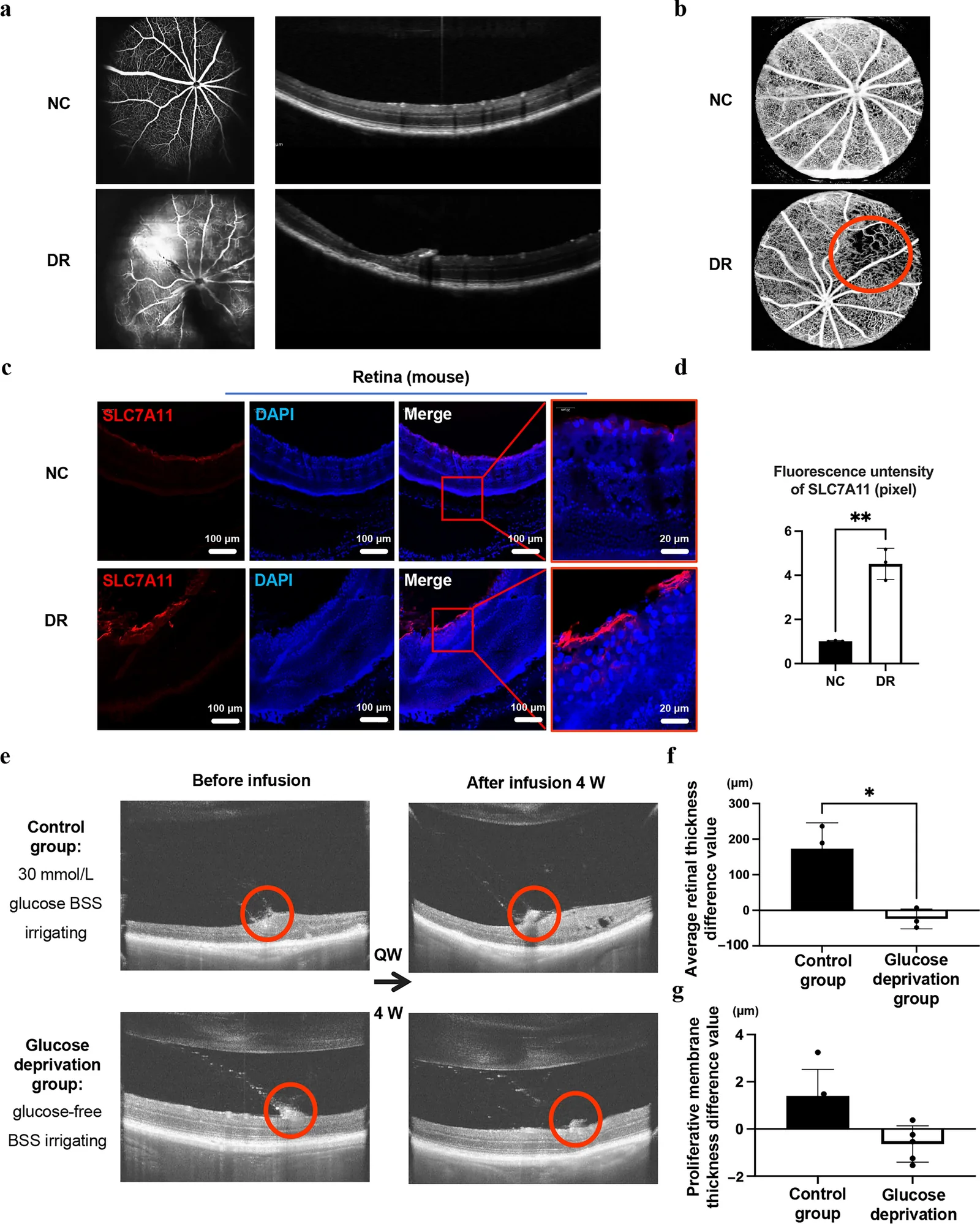
Glucose-free vitreous lavage attenuates retinal thickening in diabetic retinopathy (DR) mice. **a** Representative fluorescein fundus angiography and optical coherence tomography (OCT) images of control (NC) and DR C57 mice. The DR group showed vascular leakage and hyperreflective foci. **b** OCT angiography images from a DR mouse, confirming distinct nonperfusion areas. **c** Immunofluorescence analysis of retinal cryosections showing that *SLC7A11* expression was markedly upregulated and predominantly localized to the retinal vascular endothelium in DR mice. Scale bars = 100 μm, 20 μm (enlarged block of the merged images). **d** Bar graph showing the quantification of immunofluorescent signals from (**c**). **e** Representative OCT images after a 4-week course of weekly vitreous lavage with either a glucose-free or high-glucose solution. **f** Quantification of the changes in average retinal thickness for the groups shown in (**e**). **g** Quantification of the changes in proliferative membrane thickness difference value for the groups shown in (**e**) (red circles). BSS, balanced salt solution; DAPI, 4′,6-diamidino-2-phenylindole. *P < 0.05, **P < 0.01

To investigate the in-vivo relevance, we established a streptozotocin-induced DR mouse model in C57BL/6 mice and performed OCT and OCTA examinations. Compared with the controls, the DR group exhibited distinct nonperfusion areas, retinal edema, and detachment, with fluorescein angiography confirming vascular leakage, and OCT revealing hyperreflective regions indicative of cellular infiltration, subretinal fluid accumulation, and photoreceptor degeneration (Fig. 7a, b). Notably, while this streptozotocin-induced model successfully recapitulated early-stage DR features, such as vascular leakage, nonperfusion, and edema, it did not consistently develop structured FVMs characteristic of human PDR. Thus, our findings primarily demonstrated *SLC7A11* upregulation in pathological retinal vasculature and the principle of glucose-deprivation therapy rather than modeling advanced PDR fibrovascular proliferation. Crucially, in agreement with our in-vitro data, immunofluorescence analysis of retinal sections showed markedly upregulated *SLC7A11* expression in DR mice, predominantly localized to the retinal vascular endothelium, with a filamentous pattern along the pathological blood vessels (Fig. 7c, d).

Based on our central hypothesis that HG-adapted, *SLC7A11*-high cells are extremely vulnerable to disulfidptosis, we reasoned that inducing acute, local glucose deprivation could selectively eliminate the pathological neovascular cells driving PDR. To test this novel therapeutic strategy, we administered a 4-week treatment involving weekly vitreous lavage. The outcomes were markedly divergent: the group receiving glucose-free irrigation experienced attenuated progression of the proliferative membrane. In contrast, the control group receiving HG irrigation exhibited disease progression (Fig. 7e). To quantify this therapeutic effect, we measured the changes in average retinal thickness. The analysis showed progressive edema in the control group, with an average increase in thickness of 172.8 ± 42.0 μm (mean ± standard error of the mean). Conversely, the treatment group demonstrated a clear therapeutic benefit, with an average decrease in thickness of 24.2 ± 16.2 μm (Fig. 7f). Furthermore, the quantification of proliferative membranes corroborated these findings. While the control group showed continued thickening of the proliferative membranes, the glucose deprivation group showed a reduction in membrane thickness (Fig. 7g). These in-vivo results validated that inducing disulfidptosis via local glucose deprivation could effectively reverse the key pathological features of PDR.

In conclusion, these in-vivo findings demonstrated that *SLC7A11* is highly expressed in the pathological retinal vasculature of diabetic mice, thereby driving the progression of pathological proliferative membranes under hyperglycemic conditions. Importantly, therapeutic vitreous lavage with a glucose-free solution effectively attenuated the progression of these pathological proliferative membranes by exploiting the *SLC7A11*-mediated metabolic vulnerability through the induction of disulfidptosis.

### Chronic hyperglycemia induces a state of decompensated reductive stress in vivo, priming cells for disulfidptosis

**Fig. 8:**
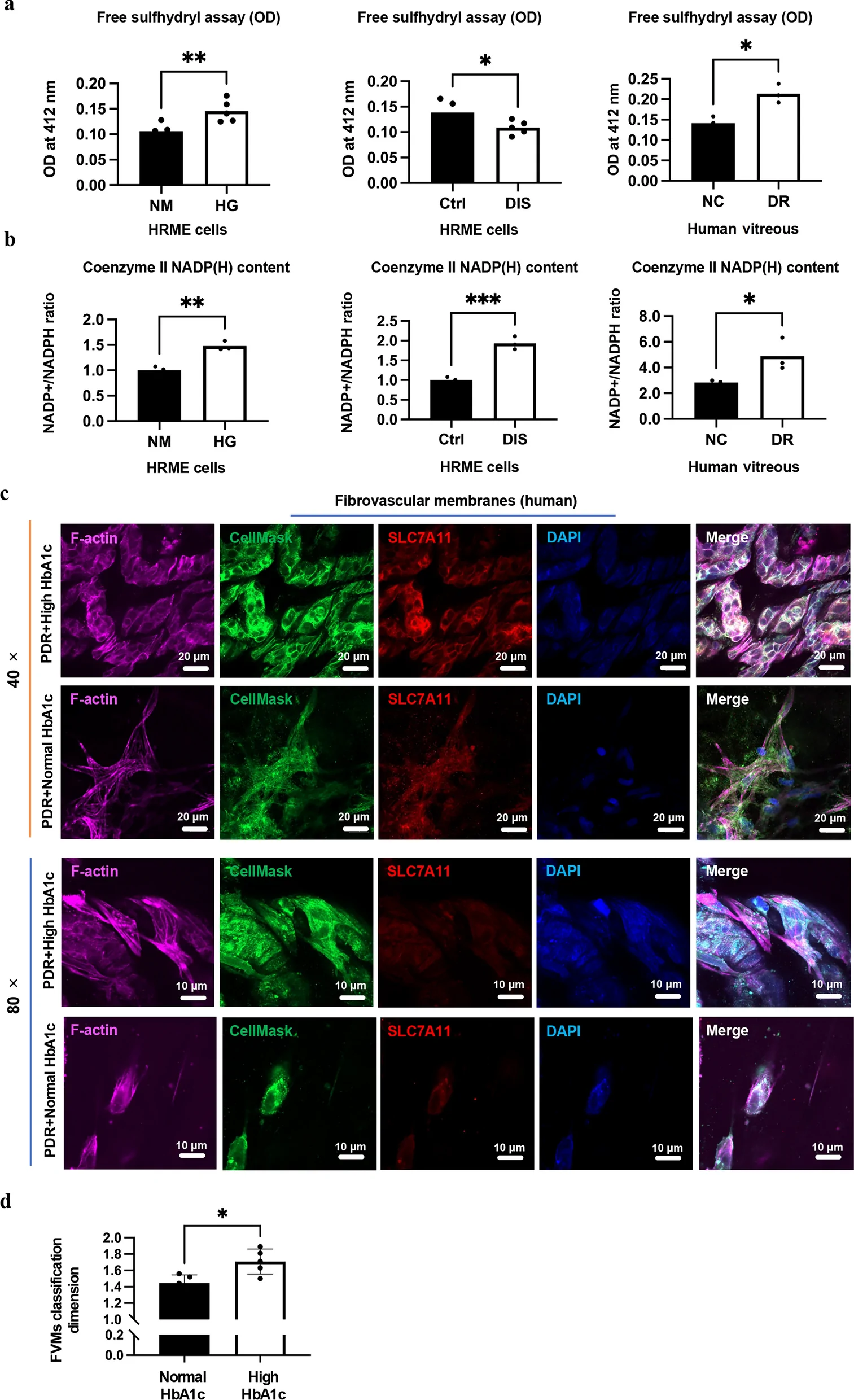
High glucose (HG) reduces antioxidant capacity and elevates oxidative stress. **a** Panel illustrating changes in free sulfhydryl content, a measure of antioxidant capacity, in human retinal microvascular endothelial (HRME) cells and human vitreous under HG or disease conditions. **b** Panel showing corresponding alterations in the NADP+/NADPH ratio, a key indicator of cellular oxidative stress, across the same experimental models and patient samples. **c** Representative immunofluorescence co-staining of SLC7A11 with CellMask and F-actin in epiretinal fibrovascular membranes (FVMs) derived from patients with proliferative diabetic retinopathy (PDR) stratified by HbA1c levels (Norm HbA1c vs. High HbA1c groups). Nuclei were counterstained with 4′,6-diamidino-2-phenylindole (DAPI; blue). **d** Quantification of the changes in fractal dimension for the groups shown in (**c**). Ctrl, control; DIS, glucose deprivation; DR, diabetic retinopathy; HbA1c, glycated hemoglobin; NM, normal medium; OD, optical density. *P < 0.05, **P < 0.01, ***P < 0.001

We found that HRMECs cultured in HG medium exhibited significant elevations in intracellular free sulfhydryl content compared with those in the normal medium control group (Fig. 8a), which was validated in vivo. Next, we measured the NADP^+^/NADPH ratio. Both HG-cultured HRMECs and vitreous humor derived from patients with DR exhibited significantly higher NADP^+^/NADPH ratios than their respective controls did (Fig. 8b).

Moreover, we tested the effects of disrupting this glucose-dependent state. When HG-adapted cells were subjected to glucose deprivation, their redox homeostasis catastrophically collapsed. With the glucose supply for NADPH regeneration severed, the free sulfhydryl pool rapidly and significantly depleted (Fig. 8a, middle panel), coupled with a massive surge in the NADP^+^/NADPH ratio to levels far exceeding those of the control group (Fig. 8b, middle panel).

Finally, immunofluorescence analysis of patient-derived FVMs directly correlated *SLC7A11* expression with clinical glycemic control (Fig. 8c). FVMs derived from patients with high HbA1c levels displayed high *SLC7A11* expression. Their F-actin cytoskeleton was organized into peripheral cortical rings, forming a stable and cohesive cellular monolayer [27, 28]. To correlate these in-vivo observations with the long-term glycemic status of patients, we analyzed FVMs derived from individuals with varying levels of glycated hemoglobin (HbA1c), a key clinical indicator of chronic blood glucose control. FVMs obtained from patients with normalized HbA1c levels exhibited markedly reduced SLC7A11 levels, and their cytoskeleton was broken down, forming loose skeletal fibers [9]. To objectively quantify the architectural remodeling of the F-actin cytoskeleton, we performed fractal dimension analysis. The results revealed that FVMs derived from patients with high HbA1c levels possessed a highly complex cytoarchitecture characteristic of a proliferative state, whereas those derived from patients with normal HbA1c levels did not (Fig. 8d).

Taken together, these data provide support for a unique mechanism. Chronic hyperglycemia, both in vitro and in the human eye, establishes a specific and vulnerable biochemical state defined by GSH depletion and NADPH oxidation. This chronic stress condition primed the cells for death. A subsequent acute insult acts as a final trigger, causing complete collapse of the already-compromised reductive system, leading to *SLC7A11*-mediated disulfidptosis.

## Discussion

Our integrated analysis, spanning bioinformatics screening to preclinical validation, revealed a dual role for the cystine/glutamate antiporter, SLC7A11, in PDR. Specifically, under hyperglycemic conditions, its high expression not only promoted a pro-angiogenic phenotype but also rendered neovascularization highly dependent on the HG microenvironment. However, upon glucose deprivation, elevated *SLC7A11* expression caused aberrant disulfide accumulation, which subsequently induced cell death, exhibiting features consistent with disulfidptosis in pathological neovascularization.

Under nondiabetic conditions, SLC7A11 primarily maintains intracellular redox balance by importing cystine for GSH synthesis. This mechanism is indispensable for protecting highly metabolically active retinal cells, particularly the retinal pigment epithelium and quiescent microvasculature, from endogenous oxidative stress and lipid peroxidation [29, 30]. However, in the context of PDR, our in vitro, in vivo, and human clinical sample analyses consistently confirmed that *SLC7A11* is significantly and specifically upregulated in the pathological endothelium. The molecular mechanism linking hyperglycemia to *SLC7A11* upregulation likely involves an integrated stress response. Chronic HG triggers mitochondrial reactive oxygen species production and endoplasmic reticulum stress, thereby activating transcription factors, such as nuclear factor erythroid 2-related factor 2 (Nrf2), activating transcription factor 4, and hypoxia-inducible factor-1α (HIF-1α), all of which are established drivers of *SLC7A11* transcription [31–35].

Crucially, our in-vitro data indicated that VEGF expression changes in parallel with *SLC7A11* knockdown and overexpression. This relationship suggests that the pro-angiogenic effect of *SLC7A11* is intrinsically linked to canonical neovascular signaling [36, 37]. Rapid endothelial proliferation and migration require substantial metabolic and antioxidant support; by heavily upregulating *SLC7A11* expression, pathological endothelial cells can sustain the high GSH levels necessary to neutralize the high reactive oxygen species levels generated during VEGF-driven angiogenesis [38–40]. Our Gene Ontology enrichment analysis revealed that the concurrent upregulation of “glucose homeostasis” and “angiogenesis” pathways supported this integrated stress response model.

However, Yuan et al. reported that the expression of *SLC7A11* decreased in the retina of DR mice [41]. This discrepancy might be attributed to numerous reasons. First, our single-cell analysis revealed that *SLC7A11* upregulation was highly cell-type-specific, being most prominent in endothelial cells within the pathological neovascular compartment. The measurement of whole retinas might mask this endothelial-specific upregulation, particularly if other cell types show decreased expression. Second, early diabetic changes might trigger *SLC7A11* downregulation due to impairment of the Nrf2 pathway. However, in established PDR, chronic oxidative stress and HIF-1α activation drive compensatory *SLC7A11* upregulation as a survival adaptation [42, 43]. Third, the interspecies divergence in biological responses could underlie the contrasting results obtained from human tissue analyses and animal studies [44].

Moreover, we observed that *SLC7A11*-OE cells, cells chronically adapted to HG, and patient-derived FVMs underwent rapid death upon acute glucose withdrawal, exhibiting the classic morphological hallmarks of disulfidptosis, including collapse of the F-actin cytoskeleton and cell shrinkage. As a key regulator of the cellular response to oxidative stress, SLC7A11 facilitates cystine uptake to support the synthesis of GSH, the primary intracellular antioxidant [9]. In the presence of ample glucose, some glucose was shunted into the pentose phosphate pathway, thereby generating the NADPH required by the thioredoxin reductase system to reduce the high influx of cystine into cysteine for GSH synthesis [45]. When glucose was removed, the NADPH supply plummeted. Neovascular cells still expressing high levels of SLC7A11 continued to import cystine, which in the absence of NADPH-dependent reduction, led to a massive accumulation of intracellular disulfides and cell death, exhibiting features consistent with disulfidptosis. Elevated levels of free thiol and NADPH under hyperglycemic conditions conferred cellular protection against oxidative stress, thus potentially facilitating metabolic adaptation to HG [46]. Glucose withdrawal converted this critical dependency into a lethal vulnerability. With the NADPH supply cut off, cells lost the capacity to reduce disulfide bonds—a failure that directly induced disulfidptosis. Disulfidptosis promotes aberrant disulfide cross-linking of actin and other cytoskeletal proteins, leading to loss of structural integrity and cell death [9]. This mechanism supports the results of our study regarding *SLC7A11*. High *SLC7A11* expression protected tumor cells and maintained antioxidant defenses but also sensitized cells to glucose deprivation-induced death in pancreatic ductal adenocarcinoma, hepatocellular carcinoma, and glioblastoma [9, 47–49].

**Fig. 9:**
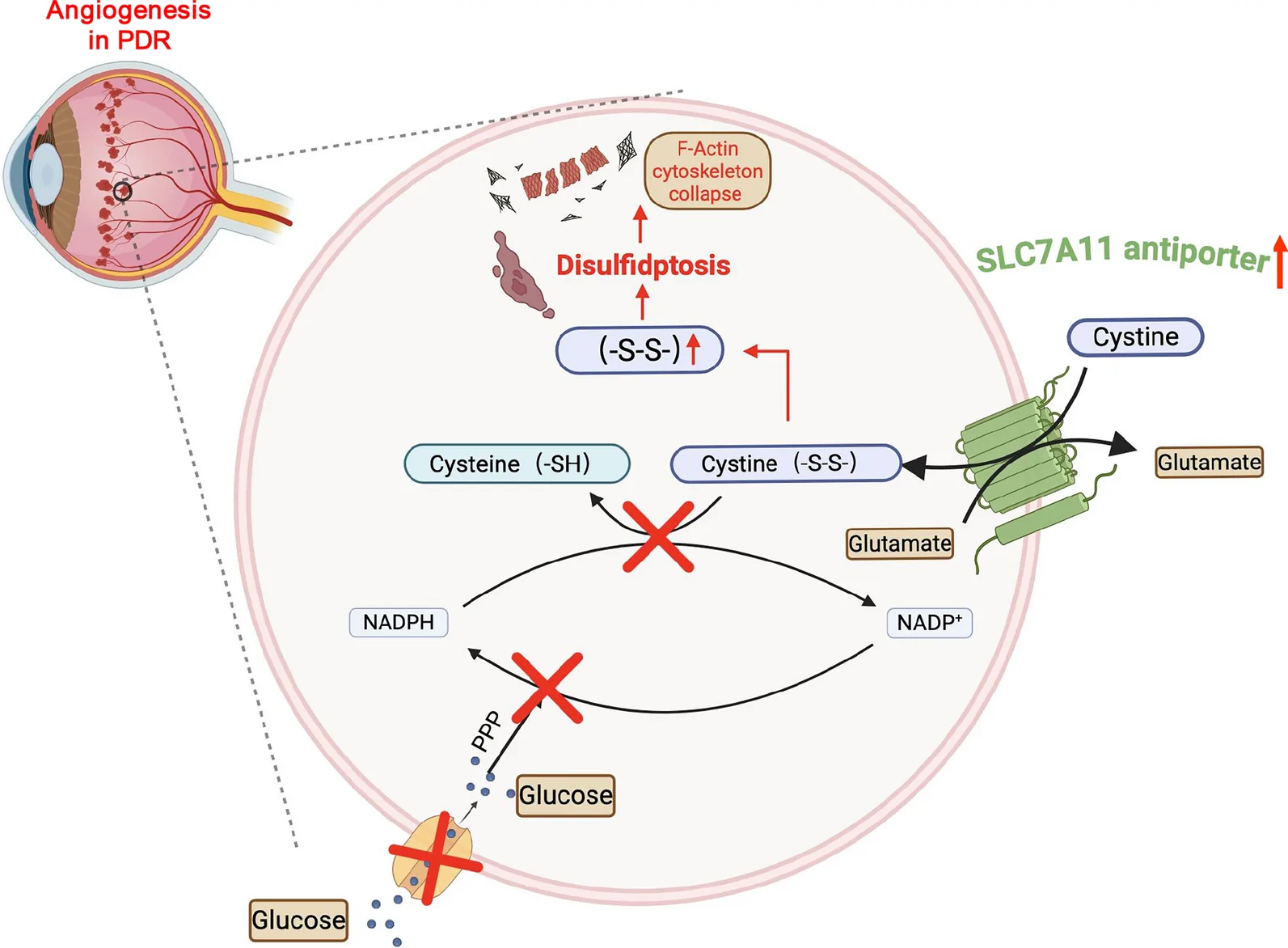
Schematic model of SLC7A11-mediated disulfidptosis in PDR pathogenesis and treatment. The red text, arrows, and crosses illustrate disulfidptosis induced by glucose deprivation. Red arrows indicate upregulation, and red crosses indicate inhibition or blockade. PPP, pentose phosphate pathway; NADPH, nicotinamide adenine dinucleotide phosphate; SLC7A11, solute carrier family 7 member 11; F-actin, filamentous actin

Moreover, as a distinct form of RCD, disulfidptosis operates alongside ferroptosis, pyroptosis, and dysregulated autophagy, collectively constituting critical pathogenic processes [50–53]. Considering *SLC7A11* is a common and important factor in both disulfidptosis and ferroptosis [54], we also examined whether cells underwent apoptosis via the ferroptosis pathway. We found that the potent ferroptosis inhibitor, Ferr-1, failed to rescue neovascular cells, further supporting the notion that the cell death pathway is consistent with disulfidptosis rather than ferroptosis in PDR under glucose deprivation. We acknowledge that definitive confirmation of canonical disulfidptosis requires the identification of actin-specific interprotein disulfide bonds via specialized biochemical assays [9], which should be investigated in future studies. Our current morphological, biochemical, and pharmacological rescue data strongly demonstrate features highly consistent with *SLC7A11*-mediated disulfidptosis. When translating this vulnerability in vivo, our preclinical vitreous lavage model demonstrated that localized glucose deprivation effectively attenuated the progression of pathological proliferative membranes. The proposed mechanisms underlying PDR progression and therapeutic intervention are illustrated in Fig. 9.

In summary, this study revealed that *SLC7A11* plays a dual role in the development and progression of PDR and that its mechanism of action is strictly dependent on ambient glucose levels. Targeting *SLC7A11*-mediated disulfidptosis may represent a promising therapeutic strategy for controlling PDR progression.

## Conclusions

In summary, our study identified *SLC7A11* as a key regulator of redox metabolism in PDR pathogenesis. We demonstrated a context-dependent dual role for *SLC7A11*: it promotes pathological angiogenesis under hyperglycemia by maintaining redox homeostasis yet paradoxically renders endothelial cells hypersensitive to glucose deprivation-induced cell death, exhibiting features consistent with disulfidptosis. Importantly, exploiting this metabolic vulnerability via localized glucose restriction effectively attenuated the progression of retinal neovascularization. Thus, our findings not only elucidate a novel *SLC7A11*-mediated mechanism but also suggest that inducing disulfidptosis could be a promising therapeutic strategy for the clinical management of PDR.

## Supplementary Information

Additional File 1. Supplementary figures and tables.

## Data Availability

The datasets used or analyzed during the current study are available from Gene Expression Omnibus. The raw sequencing data have been submitted to the NCBI BioProject database under accession number PRJNA1329159.

## Abbreviations

BSA: Bovine serum albumin
BSS: Balanced salt solution
CCK-8: Cell Counting Kit-8
DEG: Differentially expressed gene
DR: Diabetic retinopathy
DRG: Disulfidptosis-related gene
ECM: Endothelial cell medium
FBS: Fetal bovine serum
FC: Fold change
FVM: Fibrovascular membrane
GEO: Gene Expression Omnibus
GSH: Glutathione
HG: High glucose
HIF-1α: Hypoxia-inducible factor-1α
HRMEC: Human retinal microvascular endothelial cell
NADP+: Nicotinamide adenine dinucleotide phosphate (oxidized)
NADPH: Nicotinamide adenine dinucleotide phosphate
Nrf2: Nuclear factor erythroid 2-related factor 2
OCT: Optical coherence tomography
OCTA: Optical coherence tomography angiography
PBS: Phosphate-buffered saline
PDR: Proliferative diabetic retinopathy
PFA: Paraformaldehyde
RCD: Regulated cell death
RNA-seq: RNA sequencing
scRNA-seq: Single-cell RNA sequencing
siRNA: Small interfering RNA
VEGF: Vascular endothelial growth factor
VEGFA: Vascular endothelial growth factor A
