# Supplementary material for: SLC7A11-mediated disulfidptosis underlies the pathogenesis of proliferative diabetic retinopathy
Source: Eye Vis (Lond). 2026 Sep 26;13:45. doi: 10.1186/s40662-026-00511-2 (PMC13656585; doi:10.1186/s40662-026-00511-2)
Supplement: Supplementary file 1 [file 40662_2026_511_MOESM1_ESM.docx]

**Supplementary Materials**

**Supplementary Figure S1.** Schematic of the experimental design for the in vivo vitreous lavage study. In the established diabetic mouse model of proliferative retinopathy, animals were randomly assigned to one of two experimental arms. The treatment group, designated the "Glucose deprivation group", received weekly vitreous irrigation with a glucose-free balanced salt solution. The "Control group" received an identical lavage procedure using a balanced salt solution supplemented with 30 mmol/L glucose to mimic the hyperglycemic vitreous environment.

**Supplementary Figure S2.** The whole membrane images of western blots shown in Figures 2c (representative 9 out of 12 pairs are shown). DR, diabetic retinopathy

**Supplementary Fig.3:** The whole membrane images of western blots shown in Figures 3a, 4a and 4h. HG, high glucose; NM, normal medium

**Table S1. List of 14 disulfidptosis-related genes**

| **List of 14 disulfidptosis-related gene** | |
| --- | --- |
| 1 | ACTB |
| 2 | MYH10 |
| 3 | WASF2 |
| 4 | MYH9 |
| 5 | DSTN |
| 6 | FLNA |
| 7 | SLC7A11 |
| 8 | GYS1 |
| 9 | TLN1 |
| 10 | PDLIM1 |
| 11 | FLNB |
| 12 | CYFIP1 |
| 13 | RPN1 |
| 14 | IQGAP1 |

**Table S2. Antibodies for western blot and immunofluorescence**

|  | **Antibodies** | **Cat.no** | **Manufacture** | **Dilution ratio** |
| --- | --- | --- | --- | --- |
| **Western blot** | anti-SLC7A11 | DF12509 | affbiotech | 1:2000 |
|  | anti-VEGFA | AF5131 | affbiotech | 1:2000 |
|  | anti-CD31 | 553370 | [BD Biosciences](https://www.bdbiosciences.com/zh-cn) | 1:1000 |
|  | anti-β-tubulin | AF7011 | affbiotech | 1:2000 |
| **Immunofluorescence** | DAPI | C1006 | beyotime | - |
|  | anti-SLC7A11 | DF12509 | affbiotech | 1:500 |

**Table S3. Diagnosis information of patients**

| **Subject no.** | **Diagnosis** | **Gender** | **Age (years)** | **HbA1c (in the fasting state)** |
| --- | --- | --- | --- | --- |
| 1 | PDR | Male | 78 | 9.54 |
| 2 | PDR | Female | 31 | 9.93 |
| 3 | PDR | Female | 65 | 9.14 |
| 4 | PDR | Female | 77 | 8.78 |
| 5 | PDR | Male | 59 | 9.48 |
| 6 | PDR | Female | 44 | 9.48 |
| 7 | PDR | Male | 50 | 6.77 |
| 8 | PDR | Female | 55 | 5.63 |
| 9 | PDR | Male | 78 | 7.54 |
| 10 | PDR | Female | 66 | 6.25 |
| 11 | PDR | Male | 54 | 6.56 |
| 12 | PDR | Male | 40 | 7.00 |
| 1 | Epiretinal membranes | Male | 34 | 6.02 |
| 2 | Epiretinal membranes | Female | 41 | 5.62 |
| 3 | Epiretinal membranes | Male | 68 | 6.50 |
| 4 | Epiretinal membranes | Female | 50 | 5.07 |
| 5 | Epiretinal membranes | Female | 44 | 4.96 |
| 6 | Epiretinal membranes | Male | 59 | 5.35 |
| 7 | Idiopathic MH | Female | 48 | 5.32 |
| 8 | Idiopathic MH | Male | 55 | 6.22 |
| 9 | Idiopathic MH | Male | 29 | 7.00 |
| 10 | Epiretinal membranes | Female | 65 | 5.72 |
| 11 | Epiretinal membranes | Female | 46 | 5.22 |
| 12 | Idiopathic MH | Male | 55 | 5.86 |

HbA1c = glycated hemoglobin; MH = macular hole; PDR = proliferative diabetic retinopathy;
